# Management of Exciton for Highly-Efficient Hybrid White Organic Light-Emitting Diodes with a Non-Doped Blue Emissive Layer

**DOI:** 10.3390/molecules24224046

**Published:** 2019-11-08

**Authors:** Wei Luo, Xing Chen, Shuang-Qiao Sun, Yi-Jie Zhang, Tong-Tong Wang, Liang-Sheng Liao, Man-Keung Fung

**Affiliations:** 1 Jiangsu Key Laboratory for Carbon-Based Functional Materials & Devices, Institute of Functional Nano & Soft Materials (FUNSOM), Soochow University, Suzhou 215123, China; 20174214009@stu.suda.edu.cn (W.L.); 20184214124@stu.suda.edu.cn (X.C.); sun1993love@126.com (S.-Q.S.); 20174214055@stu.suda.edu.cn (Y.-J.Z.); 20174214052@stu.suda.edu.cn (T.-T.W.); lsliao@suda.edu.cn (L.-S.L.); 2 Institute of Organic Optoelectronics, Jiangsu Industrial Technology Research Institute (JITRI), 1198 Fenhu Dadao, Wujiang, Suzhou 215123, China

**Keywords:** white organic light-emitting diodes, hybrid, non-doped blue emissive layer, exciton management

## Abstract

Hybrid white organic light-emitting diodes (WOLEDs) have drawn great attention both for display and solid-state lighting purposes because of the combined advantages of desirable stability of fluorescent dyes and high efficiency of phosphorescent materials. However, in most WOLEDs, obtaining high efficiency often requires complex device structures. Herein, we achieved high-efficiency hybrid WOLEDs using a simple but efficacious structure, which included a non-doped blue emissive layer (EML) to separate the exciton recombination zone from the light emission region. After optimization of the device structure, the WOLEDs showed a maximum power efficiency (PE), current efficiency (CE), and external quantum efficiency (EQE) of 82.3 lm/W, 70.0 cd/A, and 22.2%, respectively. Our results presented here provided a new option for promoting simple-structure hybrid WOLEDs with superior performance.

## 1. Introduction

White organic light-emitting diodes (WOLEDs), which exhibit splendid properties such as high-quality white light without glare and toxic substances, fabrication flexibility, low power consumption, and high luminous efficiency, are widely regarded as a desirable solid-state light source and an indispensable layer for television and micro displays [1,2,3]. Generally speaking, WOLEDs can be classified in three ways: all-fluorescent, all-phosphorescent, and hybrid (mostly a combination of fluorescent and phosphorescent materials) [4,5]. Unlike the all-fluorescent and all-phosphorescent WOLEDs, which often suffer from low efficiency or short lifetime, the hybrid WOLED strategy has been regarded as a feasible method to obtain devices with high performance [6,7,8], as they can combine the advantages of long-lifetime blue fluorophores and high-efficiency red and green phosphors [9,10,11]. Hybrid WOLEDs can be divided into two types of device structures: the single-emissive layer (single-EML) structure and the multi-emissive layer (multi-EML) structure [4,12,13,14,15]. For single-EML hybrid WOLEDs, the triplet energy (T1) of blue fluorescent emitters should be higher than that of the phosphorescent emitters to facilitate efficient energy transfer processes when doping phosphors into the fluorophore host or when doping fluorophores and phosphors in the host together [13,16,17,18]. Moreover, to obtain ideal white light emission, the dopant concentration and co-deposition rate should be precisely controlled in single-EML hybrid WOLEDs; otherwise, the electroluminescence (EL) spectra of organic light-emitting diodes (OLEDs) would be affected by a slight variation in the dopant concentration and hence lead to a decrease of device efficiency [14,17]. To overcome the disadvantages of single-EML hybrid WOLEDs, an alternative choice is to apply a multi-EML hybrid structure, which has many great features such as flexibility in manipulating each EML so as to manage the exciton distributions in different EMLs in a controlled manner, making it easier to achieve high efficiency than the single-EML hybrid WOLEDs [19,20,21].

In the past years, multi-EML hybrid WOLEDs have been widely developed. Forrest et al. reported the first efficient multi-EML hybrid WOLED in 2006 with a maximum power efficiency (PE) of 22.1 lm/W and an external quantum efficiency (EQE) of 11.0% by employing an interlayer between the phosphorescent and fluorescent EMLs [1]. In 2012, Ma et al. developed a new way to construct the interlayer. The hybrid WOLEDs exhibited a maximum PE, current efficiency (CE) and EQE of 34.2 lm/W, 29.4 cd/A, and 13.8%, respectively [22]. Although the interlayers are generally used for preventing mutual exciton quenching in the multi-EML hybrid WOLEDs devices, the introduction of interlayers also bring additional heterojunction interfaces between layers, which would result in a higher operation voltage and lower power efficiency [23,24,25,26]. Therefore, if all excitons are manipulated and utilized well in the multi-EML hybrid WOLEDs without interlayers, the impact of the exciton quenching phenomenon will be greatly reduced. Through this strategy, the device efficiency can be enhanced and the device structure can be simplified [18,22,24].

Recently, Zhao et al. applied a non-doped EML in phosphorescent OLED devices [27]. It has been shown that by introducing a non-doped pure organic EML in hybrid WOLEDs, this device can gain higher efficiency than that of the conventional host-guest doped device. Tan et al. demonstrated highly efficient WOLED structure with non-doped ultra-thin EMLs to investigate the spatial distribution of excitons [28]. Such non-doped devices allow us easily to optimize and construct EML independently [22,29,30]. However, only a few researchers focus on the utilization of this promising structure in hybrid WOLEDs.

In this work, we designed a novel device architecture to obtain high-efficiency hybrid WOLEDs. The white light emission derives from two emitting layers: the non-doped fluorescence dye *N*,*N*′-di-(1-naphthalenyl)-*N*,*N*′-diphenyl-[1,1′:4′,1″:4″,1‴-quaterphenyl]-4,4‴-diamine (4P-NPD) as the blue EML, and the efficient phosphorescent material bis(4-phenylthieno[3,2-c]pyridine) (acetylacetonate)iridium(III) (PO-01) doped in 4P-NPD to form the orange EML. We separated the light emission zone of complementary colors from exciton recombination zone deliberately, and then regulated excitons recombination to balance the carrier injection and reduced the exciton quenching to achieve high-efficiency OLED. The resulting hybrid WOLED showed a maximum PE, CE, and EQE of 82.3 lm/W, 70.0 cd/A and 22.2%, respectively, and still had 77.5 lm/W, 68.0 cd/A and 21.6%, respectively, at a luminance of 100 cd/m^2^. It is suggested that the dopant concentrations, the thickness of the non-doped EML and even different host materials play critical roles in determining the performance of the WOLEDs, and the high-performance in this multi-EML system is derived from the efficient energy transfer from the non-doped fluorescence layer to the phosphorescent dopant layer.

## 2. Results and Discussion

**Figure 1:**
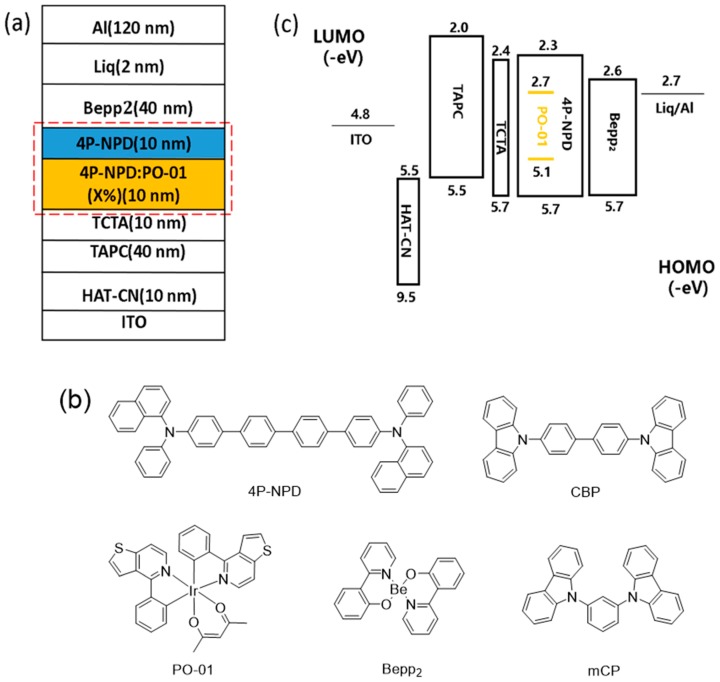
(**a**) Schematic structure of the white organic light-emitting diode (WOLED). (**b**) Chemical structure of organic materials used in the WOLED. (**c**) Energy level diagram of the WOLED. ITO: indium tin oxide; HAT-CN: dipyrazino [2–f:2′,3′-h] quinoxaline-2,3,6,7,10,11-hexacarbonitrile; Liq: lithium quinolate; TCTA: *N*,*N*,*N*-tris(4-(9-carbazolyl)phenyl)amine; 4P-NPD: *N*,*N*′-di-(1-naphthalenyl)-*N*,*N*′-diphenyl-[1,1′:4′,1″:4″,1‴-quaterphenyl]-4,4‴-diamine; TAPC: 1,1-Bis-(4-methylphenyl)-aminophenyl)-cyclo-hexane; PO-01: bis(4-phenylthieno[3,2-c]pyridine) (acetylacetonate)iridium(III); Bepp2: Bis[2-(2-hydroxyphenyl)-pyridine]-beryllium; HOMO: highest occupied molecular orbital; LUMO: lowest unoccupied molecular orbital.

We first investigated the effect of the dopant concentration on device efficiency. As depicted in Figure 1a, the hybrid WOLED device has a configuration of indium tin oxide (ITO)/dipyrazino [2–f:2′,3′-h] quinoxaline-2,3,6,7,10,11-hexacarbonitrile (HAT-CN; 10 nm)/1,1-Bis-(4-methylphenyl)-aminophenyl)-cyclo-hexane (TAPC; 40 nm)/*N*,*N*,*N*-tris(4-(9-carbazolyl)phenyl)amine (TCTA; 10 nm)/4P-NPD: x wt% PO-01 (10 nm)/4P-NPD (10 nm)/Bis[2-(2-hydroxyphenyl)-pyridine]-beryllium (Bepp2; 40 nm)/ lithium quinolate (Liq; 2 nm)/Al (100 nm). Dipyrazino [2–f:2′,3′-h] quinoxaline-2,3,6,7,10,11-hexacarbonitrile (HAT-CN) was used to form a hole-injecting layer (HIL). 1,1-Bis-(4-methylphenyl)-aminophenyl)-cyclo-hexane (TAPC) and *N*,*N*,*N*-tris(4-(9-carbazolyl)phenyl)amine (TCTA) were used as the hole-transport layer (HTL). A blue fluorescence material 4P-NPD and orange phosphorescent material PO-01 were applied as the EMLs. Bis[2-(2-hydroxyphenyl)-pyridine]-beryllium (Bepp2) and lithium quinolate (Liq) were utilized as the electron-transport layer (ETL) and electron-injection layer (EIL), respectively. Indium tin oxide (ITO) and aluminum (Al) were utilized as the anode and cathode, respectively. Here, we selected x = 0.5, 1, 5, and 8 as the dopant concentrations. The orange EML is phosphorescent material PO-01 doped in a blue fluorescence material 4P-NPD and another non-doped 4P-NPD is the blue EML. Figure 1b depicts the molecular structures of 4P-NPD, PO-01, and Bepp2. As a deep-blue fluorescent emitter, 4P-NPD shows an emission peak of 428 nm and photoluminescence quantum yield of 92%. Bepp2 often serves as the ETL in blue OLED due to its excellent electron transporting property with a mobility of 10^−4^ cm^2^ V^−1^ s^−1^ [31,32,33,34,35]. Miao et al. reported an efficient deep-blue OLED combining emissions from 4P-NPD and Bepp2. The device shows an emission peak at 432 nm and the EQE of the device was up to 5.07% [36]. These superior characteristics make them have great potential in constructing high-performance white OLEDs. The energy level diagram for each material used in this WOLED is shown in Figure 1c, in which the highest occupied molecular orbitals (HOMOs) of TAPC (5.5 eV), TCTA (5.7 eV), and 4P-NPD (5.7 eV) are similar [37,38], and therefore holes are easily injected into the vicinity of 4P-NPD/Bepp2 interface. Then the holes combine with the electrons in Bepp2, generating singlet excitons for blue emission in the non-doped blue EML, and triplet excitons through Dexter energy transfer to the phosphorescent dopants PO-01 for orange emission. This barrier-free architecture may be beneficial for promoting the EL performance of the devices.

**Figure 2:**
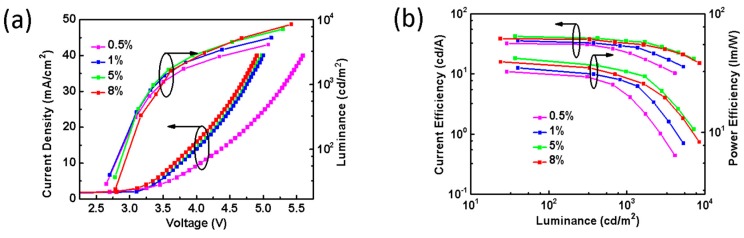
(**a**) Current density-voltage-luminance curves of the devices. (**b**) Current efficiency–luminance–power efficiency characteristics.

The concentration effect of PO-01 on the device performance is unveiled in Figure 2a. It shows that 1 wt%, 5 wt%, and 8 wt% of PO-01 have similar current density and luminance when they are at the same driving voltage. However, the current efficiency-luminance-power efficiency curves in Figure 2b indicate that when the doping concentration of PO-01 was increased from 0.5 wt% to 5 wt%, the maximum CE and PE increased from 32.1 cd/A to 42.0 cd/A and 34.5 lm/W to 45.2 lm/W, respectively. When 8 wt% of PO-01 is used, the maximum efficiencies of the device dropped to 38.5 cd/A and 39.3 lm/W. At low concentration (e.g., 0.5 wt%), there is an absence of concentration quenching effect; however, it causes incomplete energy transfer in the host–guest system. On the contrary, high concentration of PO-01 may cause exciton quenching [1,11,27]. Therefore, our results suggest that the device with 5 wt% of PO-01 is the most effective to reduce the non-radiative decay from the triplet excitons and hence the efficiency of the device can be enhanced. From these data we conclude that the device efficiencies vary with the doping concentrations, and the optimized dopant concentration leads to efficient utilization of excitons for EL emission with good device performance.

**Figure 3:**
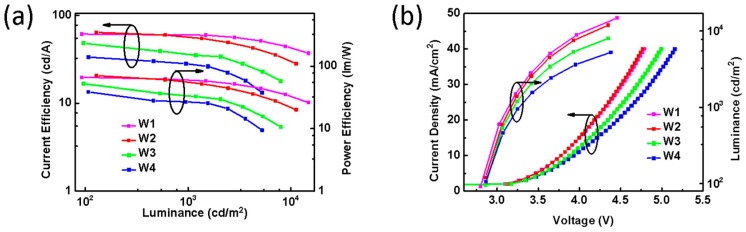
(**a**) Current efficiency–luminance–power efficiency characteristics of W1, W2, W3, and W4. (**b**) Current density–voltage–luminance curves of those devices.

Secondly, we studied the effect of thickness ratio between the non-doped blue EML and the orange EML on the chromaticity and device efficiency. We designed the following device structure: ITO/HAT-CN (10 nm)/TAPC (40 nm)/TCTA (10 nm)/4P-NPD: 5 wt% PO-01 (20-Y nm)/4P-NPD (Y nm)/Bepp2 (40 nm)/Liq (2 nm)/Al (100 nm), where Y = 1, 5, 10, and 15, and the corresponding devices are named as W1, W2, W3, and W4, respectively. Figure 3a shows that the maximum CE and PE of W1 and W2 are 66.6 lm/W, 70.6 lm/W and 61.2 cd/A, 64.0 cd/A, respectively. Their Commission International de I’Eclairage (CIE) coordinates (x, y) as shown in Table 1 are (0.48, 0.49) and (0.46, 0.46), respectively, indicating those devices with warm white emission. By increasing the thickness of the non-doped 4P-NPD layer, the maximum CE and PE of W3 and W4 reduced to 47.6 lm/W, 38.7 lm/W and 42.4 cd/A, 33.5 cd/A, respectively, and their CIE (x, y) values indicate that the WOLEDs have an increased blue emission. Figure 3b is the current density-voltage-luminance characteristics of these devices. It is clearly seen that W1 and W2 have higher current density and luminance than W3 and W4, indicating that W1 and W2 may achieve a better balance in energy transfer and charge transport for efficient WOLEDs compared to W3 and W4.

**Figure 4:**
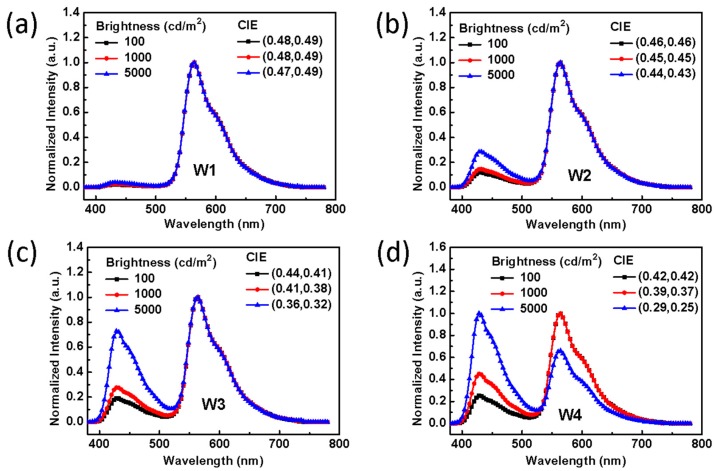
The normalized electroluminescence (EL) spectra and corresponding CIEs of (**a**) W1, (**b**) W2, (**c**) W3, and (**d**) W4 at luminance of 100, 1000, and 5000 cd/m^2^.

Furthermore, according to the EL spectra depicted in Figure 4a–d, we can find that the four WOLEDs exhibit different spectral stability with increasing luminance. For W1, the EL spectra show extremely stable CIE coordinates of (0.48, 0.49)/(0.48, 0.49)/(0.47, 0.49) at a luminance of 100, 1000, and 5000 cd/m^2^, respectively, with a variation of only (±0.01, ±0.00). Device W2 also has good stable CIE coordinates of (0.46, 0.46)/(0.45, 0.45)/(0.44, 0.43) with Δ(x, y) of (±0.02, ±0.03). On the contrary, the EL spectra of devices W3 and W4 are instable with increasing brightness as the blue emission is obviously enhanced, with the result that W3 and W4 have Δ(x, y) of (±0.08, ±0.09) and (±0.13, ±0.17) with increasing device luminance to 5000 cd/m^2^. In addition, the CIE of W2 is closer to the warm white emission point (0.45, 0.41) in the CIE Chromaticity Diagram than that of W1. From this data, we suggest that the energy transfer process under different thickness ratio between the orange EML and blue EML may be responsible for the differences of these devices efficiencies and CIE coordinates. There are three energy transfer processes: (1) excitons are generated in the interface of 4P-NPD and Bepp_2_; (2) then singlet excitons are used by the non-doped 4P-NPD layer through the Foster transfer channel to form fluorophore; and (3) the triplet excitons are transferred from the blue EML to the orange EML by Dexter transfer. From the EL spectra of the devices W1 to W3, as increasing the brightness, the orange light intensity was unchanged, substantiating that all triplet excitons can be utilized by the phosphorescent material PO-01. However, the blue light intensity substantially increases when we increase the brightness of the devices from 100 to 5000 cd/m^2^, because more singlet excitons are consumed in the non-doped 4P-NPD layer at high current density. Also, when the devices are driven at the same brightness, the blue light intensity increases with increasing the thickness of the 4P-NPD layer because the thicker film leads to broadened blue emission zone and efficient utilization of singlet excitons. Unfortunately, even though the increase of the non-doped blue EML thickness could enhance the blue light intensity, too much blue emission is not beneficial to the device performance [39,40]. This is because the triplet diffusion length becomes large and fewer triplets can be transferred to the orange EML when the 4P-NPD layer is thick, which is the reason for the low luminous efficiencies of W3 and W4. Especially for W4, the improper thickness ratio of the blue EML to the orange EML leads to a poor exciton management when the brightness is increased, resulting in reduced orange emission and a dramatic drop in device efficiency.

**Figure 5:**
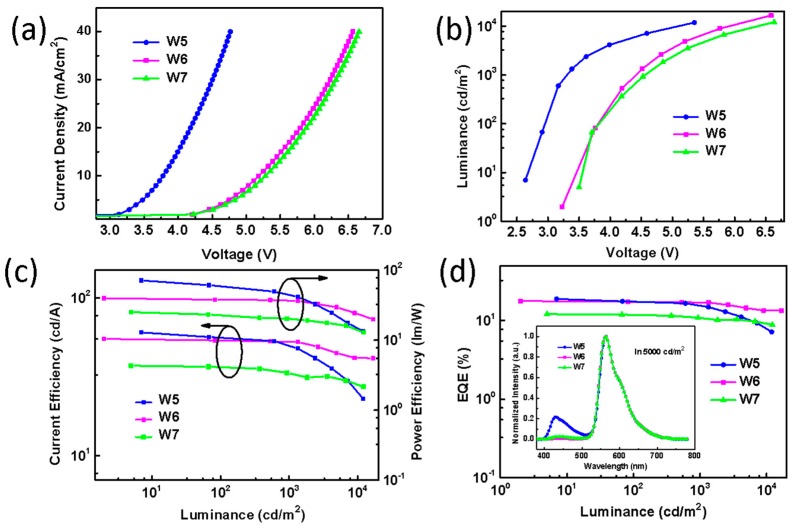
(**a**) The current density–voltage curves of W5 (4P-NPD), W6 (CBP), W7 (mCP). (**b**) Luminance-voltage curves of W5, W6, W7. (**c**) The current efficiency–luminance–power efficiency characteristics of them. (**d**) EQE: the luminance characteristics. Inset: EL spectra of W5, W6, W7 at the luminance of 5000 cd/m^2^.

Then, we fabricated three additional devices using hosts with different triplet energies to illustrate the influence of the host materials. The device structure is ITO/HAT-CN (10 nm)/TAPC (40 nm)/TCTA (10 nm)/host: 5 wt% PO-01 (15 nm)/4P-NPD (5 nm)/Bepp2 (40 nm)/Liq (2 nm)/Al (100 nm). Hosts are 4P-NPD, 4,4′-bis(carbazol-9-yl)biphenyl (CBP) and 1,3-bis(carbazol-9-yl)benzene (mCP). Their corresponding triplet energies are 2.3 eV, 2.6 eV, and 2.9 eV [21,28,36,40], and the devices are named as W5, W6, and W7, respectively. Here, we define the triplet energy difference between the hosts (4P-NPD, CBP, mCP) and guest (PO-01, T1 = 2.2 eV) is ∆, and the ∆ is 0.1 eV, 0.4 eV, and 0.7 eV, respectively. It can be seen in Figure 5a and b that the device W5 using 4P-NPD as the host has a clear distinction in term of current density and luminance compared to W6 and W7 when they were driven at the same voltage (such as 5 V). It is suggested that ∆ would influence the carrier transport and energy transfer process. Also, ∆ can form triplet exciton traps with different depths on phosphor dyes [41,42,43]. As ∆ is smaller, the triplet exciton traps effect becomes weaker and the energy loss of the triplet transfer process is reduced to help promote device efficiency. Figure 5c,d show the corresponding efficiency data. Device W5 has a maximum CE, PE, and EQE of 60.5 cd/A, 71.3 lm/W, and 18.9%, while the values of W6 and W7 are 55.0 cd/A, 39.7 lm/W, and 17.8%, and 37.2 cd/A, 25.1 lm/W, and 12.1%, respectively. Their EL spectra driven at a luminance of 5000 cd/m^2^ are shown in the inset of Figure 5d. There is an extremely weak blue emission for W6 and W7. This is probably due to the fact that the deeper HOMO of CBP (6.0 eV) and mCP (5.9 eV) would shift the exciton recombination zone to the TCTA/4P-NPD: PO-01 interface, leading to a decrease of blue emission [20,40]. Their unmatched energy levels also cause an increase of turn-on voltage for W6 and W7. Therefore, the above results suggest that the choice of host materials have a significant impact on device performance.

**Figure 6:**
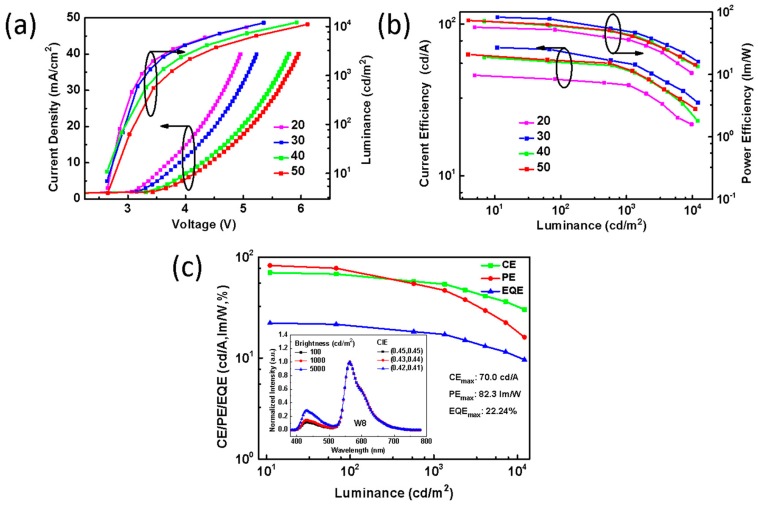
(**a**) Current density-voltage-luminance curves of four devices in this study. (**b**) Current efficiency-luminance-power efficiency characteristics with different ETL thicknesses. (**c**) CE, EQE, and PE of device W8. Inset: EL spectra and CIE of W8 at luminance of 100, 1000, and 5000 cd/m^2^.

In order to further optimize the device structure, four devices with different thicknesses of ETL were fabricated. The thickness of ETL is 20, 30, 40, and 50 nm, respectively. Figure 6a is the current density-voltage-luminance characteristics. It can be seen that the current density and luminance decrease with increasing the ETL thickness. It is believed that thicker ETL may affect the charge transport balance [44]. Figure 6b depicts the current efficiency–luminance–power efficiency curves, in which the optimal ETL thickness that obtained the highest device efficiency is 30 nm (defined as device W8), indicating that excitons are utilized most efficiently. Varying the thickness of ETL would cause the shifting of exciton recombination zone position. The luminous efficiencies of W8 is replotted in Figure 6c and listed in Table 2. It can be seen that the device W8 has a maximum forward-viewing PE, CE, and EQE of 82.3 lm/W, 70.0 cd/A, and 22.2%, respectively, without using light out-coupling technique and unstable phosphorescent blue emitter, and the efficiencies can be maintained at 77.5 lm/W, 68.0 cd/A, and 21.6%, respectively, at 100 cd/m^2^. The inset of Figure 6c indicates that W8 has a fairly good color stability, with Δ(x, y) equal to (±0.03, ±0.04) for the device brightness, increasing to 5000 cd/m^2^. The present study thus unveils a simple hybrid WOLED structure with high efficiency.

## 3. Conclusions

In summary, we have designed a simple structure by introducing a non-doped blue fluorescence emitting layer and combined it with an orange phosphor as the dopant to obtain high-performance hybrid WOLEDs. This structure acquires a good carrier balance through controlling the dopant concentration and regulating the thickness ratio between the orange EML and blue EML. In addition, by selecting appropriate host materials and optimizing the thickness of ETL, we can effectively utilize the excitons and hence the exciton quenching could be greatly reduced, resulting in high device efficiency. Our WOLED shows a maximum PE, CE, and EQE of 82.3 lm/W, 70.0 cd/A, and 22.2%, respectively, which remains at 77.5 lm/W, 68.0 cd/A, and 21.6% at the luminance of 100 cd/m^2^. The structural design of this work is simple but the device performance is superior.

## 4. Materials and Methods

### 4.1. Materials

All materials were directly purchased from commercial suppliers and used without further purification.

### 4.2. Device Fabrication and Measurements

The ITO glass substrates with a sheet resistance of 15 Ω per square were firstly cleaned with acetone, ethanol, and deionized water. They are then dried in an oven at 100 °C for 5 h and treated by ultraviolet ozone for 15 min. All the layers were thermally evaporated at a vacuum of 2 × 10^−6^ Torr. The deposition rates of HIL, HTL, EML, ETL, EIL, and Al were 0.2, 2 (for HTL, EML, ETL), 0.2, and 6 A/s, respectively, and were monitored by quartz crystals. The emissive area of the devices was 3 × 3 mm^2^. The EL spectra, luminance, and CIE coordinates of devices were measured by using Spectrascan PR655 photometer (JADAK, Syracuse, NY, USA) and Keithley 2400 source meter (Tektronix, Beaverton, OR, USA). EQEs were calculated from the corresponding current density, luminance, and EL spectra.

## Figures and Tables

**Table 1:** Summary of voltages, power efficiency (PE), current efficiency (CE), external quantum efficiency (EQE), Commission International de I’Eclairage (CIEs) coordinates, and color temperature (CCT) of the devices for W1 to W4. ^a), b)^ Voltages were measured corresponding to the onset and 1000 cd/m^2. c)^ CIE coordinates and CCT values were measured at 100 cd/m^2^.

WOLEDs	Voltage		PE (lm/W)	CE (cd/A)	EQE (%)	CIE ^c)^	CCT ^c)^ (K)
	(V) ^a)^	(V) ^b)^	Maximum/At 100 cd/m^2^			(x, y)	
W1	2.79	3.22	66.6/58.1	61.2/59.5	19.3/18.2	0.48, 0.49	2951
W2	2.84	3.27	70.6/52.3	64.0/54.5	20.3/17.4	0.46, 0.46	3131
W3	2.86	3.35	47.6/32.6	42.4/35.2	14.5/12.1	0.44, 0.41	3221
W4	2.86	3.25	38.7/26.9	33.5/28.2	11.7/9.8	0.42, 0.42	3315

**Table 2:** Summary of voltages, power efficiency, current efficiency, EQE, CIE, and CCT for devices W5 to W8. ^a), b)^ Voltages were measured corresponding to the onset and 100 cd/m^2^. ^c)^ CIE and CCT were tested at 100 cd/m^2.^

WOLEDs	Voltage		PE (lm/W)	CE (cd/A)	EQE (%)	CIE ^c)^	CCT ^c)^ (K)
	(V) ^a)^	(V) ^b)^	Maximum/At 100 cd/m^2^			(x, y)	
W5	2.63	2.90	71.3/61.0	60.5/56.5	18.9/17.6	0.45, 0.45	2885
W6	3.23	3.76	39.7/37.9	55.0/53.8	17.8/17.4	0.48, 0.48	2941
W7	3.50	3.71	25.1/23.2	37.2/36.6	12.1/11.9	0.48, 0.48	2861
W8	2.63	2.88	82.3/77.5	70.0/68.0	22.2/21.6	0.45, 0.45	3137
